# Preparation, characterization and in vitro evaluation of atorvastatin nanosuspensions

**DOI:** 10.1371/journal.pone.0335024

**Published:** 2025-10-21

**Authors:** Yaman Ahmad Akour, Ahmad Aljaberi, Saja H. Hamed, Alaa Altaher, Eman M. Migdadi

**Affiliations:** 1 Department of Pharmaceutical Sciences and Pharmaceutics, Faculty of Pharmacy, Applied Science Private University, Amman, Jordan; 2 Department of Pharmaceutics and Pharmaceutical Technology, Faculty of Pharmaceutical Sciences, The Hashemite University, Zarqa, Jordan; Queen's University Belfast, UNITED KINGDOM OF GREAT BRITAIN AND NORTHERN IRELAND

## Abstract

Poorly water-soluble drugs present a significant challenge for pharmaceutical development, particularly affecting the pharmacokinetics of orally administered drugs due to their poor dissolution. This study aimed to enhance the dissolution of a low water solubility drug atorvastatin using nanosuspension technology. Antisolvent technique was utilized to prepare atorvastatin calcium nanosuspensions. Different stabilizers were used including Cremophor, HPMC, pluronics (F127, F108, and F68), PEG400, PEG600, PEG 1k, PEG8k, PVA, PVP k30, PVP k10, PVP 44k, SLS, sodium alginate, Tween 20, and Tween 80. The prepared nanosuspensions were lyophilized using mannitol or trehalose as a lyoprotectant. Several optimum formulations were obtained. The selected best optimum formulation was 2% pluronic F127, 80 mg mannitol, and 2 min sonication time. It exhibited a mean particle size of 54.5 nm, a zeta potential of −0.809, and 0.141 PDI after reconstitution. The crystalline state of the nanoparticles was evaluated using differential scanning calorimetry (DSC) and X-ray diffraction (XRD). Fourier-transform infrared spectroscopy (FTIR) was used to assess interactions between the drug substance and the additives. In vitro drug release study was conducted for lyophilized nanosuspension in comparison to the innovator product Lipitor® in two different media, 0.05M potassium phosphate buffer pH 6.8 and 0.1N HCL. The XRD analyses indicated that the lyophilized nanosuspension was partially crystalline with some amorphization and this confirmed by DSC. FTIR results suggested that there was a physical interaction between atorvastatin and the additives. The polymer and lyoprotectant successfully preserved and coated the drug nanoparticles. The lyophilized nanosuspension exhibited superior dissolution in 0.1N HCl. However, the innovator was faster in 0.05 M phosphate buffer. In conclusion, successful preparation of lyophilized nanosuspension of atorvastatin calcium was achieved. The current findings revealed that excipient functionality in terms of stabilization, effect on solid state properties, and drug release were critical for development of stable and efficient nanosuspension.

## 1. Introduction

The pharmaceutical industry struggles with poor aqueous solubility of drugs and thus, their limited bioavailability [1] (reference). Solubility is crucial in pharmaceuticals, with 75–90% of drug candidates under development, and up to 40% of marketed products are poorly soluble [1]. This results in low bioavailability, reduced therapeutic effects, and dosage escalation that necessitate careful consideration in product design and manufacturing. To date, various strategies regarding low solubility problem have been studied include micronization, solid dispersion and supercritical fluid (SCF). Other approaches include hydrotropy, co-solvency, micellar solubilization and cryogenic technique. Additionally, methods like inclusion complex formation, nanosuspension, solid lipid nanoparticles, and nanogels have been explored [1] (reference). Among several techniques, nanosuspension was shown to be a promising and efficient strategy for enhancing the dissolution of poorly soluble drugs [1]. Nanosuspension is generally composed of biphasic colloid particles distributed in an aqueous medium with a size range of less than 1μm and stabilized by the addition of appropriate surfactants or polymers [2]. According to the Noyes-Whitney equation, increasing the surface area of drug particles leads to improving the rate of dissolution. Therefore, So, developing formulations of nanosuspension can overcome the problems associated with poorly water-soluble and lipid-soluble drugs coupled with inadequate drug delivery by enhancing their absorption and bioavailability [3]. It can also alter the pharmacokinetics of a drug and boosting its safety and efficacy [4]. Consequently, as the number of drugs with solubility and bioavailability challenges increases, this method will become progressively essential [3].

**Fig 1:**
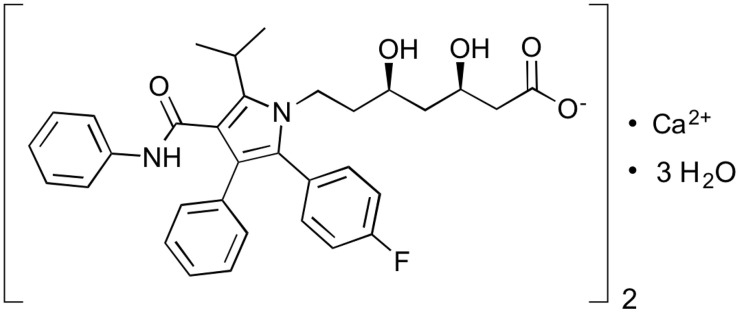
Atorvastatin calcium chemical structure.

Atorvastatin is a cholesterol-lowering drug used to reduce the risk of stroke, heart attack, and other cardiovascular diseases [5]. Atorvastatin (ATV) is a class II drug according to the Biopharmaceutical Classification System (BCS) with a low water solubility of about (0.63 mg/L). At physiological intestinal pH, atorvastatin has a high intestinal permeability. However, it has been reported that atorvastatin’s absolute bioavailability after a 40 mg oral dosage was just 12%. The limited systemic availability is related to low dissolution, pre-systemic clearance in the gastrointestinal mucosa, and hepatic first-pass metabolism [6]. Atorvastatin can be absorbed quickly after oral administration and reaches maximal concentration within 1–2 hours. The structure of atorvastatin is shown in Fig 1 and its solubility in different solvents is shown in Table 1.

**Table 1:** The solubility of atorvastatin calcium in different solvents [7–9].

Media/ pH	Solubility
0.1N HCL	0.0315 mg/ml
pH 4.5	0.0570 mg/ml
pH 5.5	0.1701 mg/ml
pH 6.8	0.2838 mg/ml
Water (pH 7.12)	0.8 mg/ml (very slightly soluble)
Ethanol	21 mg/ml (slightly soluble)
1:9 solution of DMF and Phosphate Buffered Saline (PBS) (pH 7.2)	0.1 mg/ml
Methanol	232.8 mg/ml
Acetonitrile	14.9 mg/ml

The aim of this study was to formulate atorvastatin calcium nanosuspension by the use of solvent- antisolvent precipitation technique. An attempt to enhance atorvastatin solubility and improve dissolution rate.

## 2. Materials and methods

### 2.1. Materials

Atorvastatin calcium was gifted from RAM Pharmaceuticals Ind. Co., Amman, Jordan. Acetonitrile was purchased from Scharlau, Barcelona, Spain. Hydroxypropyl methylcellulose (HPMC) was obtained from Ashland Industries Europe GmbH, Switzerland. Mannitol and Trehalose were supplied by GenoChem, Valencia, Spain. Methanol ≥ 99.9% was provided by Honeywell, France. Pluronic® F108, F68, and F127 were purchased from BIOSYNTH Carbosynth, California, USA. Polyethylene Glycol (PEG400, PEG600, PEG 1k, and PEG 8k) were supplied by IMP, Benoni, South Africa. Polyvinyl Alcohol (PVA) was obtained from Analyt analytical reagent gcc, Al Dammam, Saudi Arabia. Polyvinylpyrrolidone (PVP K10, PVP K30, and PVP 44K) were sourced from Super SC Chem, Gujarat, India. Polyvinylpyrrolidone (PVP K30 LR) was obtained from CDH fine chemicals, New Delhi, India. Sodium Lauryl Sulfate (SLS) and Sodium Alginate were purchased from Sigma-Aldrich, Central West End, USA. Polysorbate 20 (Tween 20) was sourced from MTSS, Amman, Jordan. Polysorbate 80 (Tween 80) was obtained from BBC Chemical for lab, Torre Boldone BG, Italy. Cremophor was obtained from RH (Kolliphor® RH) BASF SE/Ludwigshafen, Germany. All the chemicals and reagents were of analytical grade.

### 2.2. Preparation of atorvastatin calcium nanosuspensions using precipitation method

Atorvastatin nanosuspension was prepared via anti-solvent precipitation. A total of 20 mg of atorvastatin was dissolved in 1 mL of methanol to form the drug solution. The anti-solvent phase was prepared by dissolving a stabilizer in deionized water at three different concentrations (0.5%, 1%, and 2% w/v). Different stabilizers were used including Hydroxypropyl methylcellulose (HPMC), Pluronics® F108, F68, and F127 Polyethylene Glycol (PEG400, PEG600, PEG 1k, and PEG 8k). In addition, Polyvinyl Alcohol (PVA), Polyvinylpyrrolidone (PVP K10, PVP K30, and PVP 44K), Polyvinylpyrrolidone (PVP K30 LR), Sodium Lauryl Sulfate (SLS) and Sodium Alginate, Polysorbate 20 (Tween 20), Polysorbate 80 (Tween 80) and Cremophor.

For each formulation, 4 mL of a stabilizer solution was used. The atorvastatin solution was then introduced dropwise into the anti-solvent phase, followed by immediate sonication using a probe sonicator (Hielscher, Teltow, Germany) at 80% amplification. Sonication was carried out for either 2 or 3 minutes, with a pulse cycle of 10 seconds on and 5 seconds off. To prevent overheating during sonication, the temperature of the stabilizer solutions was maintained at 5°C ± 3°C using an ice bath. After sonication, the resulting nanosuspension was stirred at room temperature under a fume hood for 1 hour to allow complete evaporation of methanol. The resultant formulation was exposed for subsequent analysis and characterization of particle size and zeta potential using A Zetasizer (Malvern Instruments, Malvern, UK). Then the formulations with the particle size less than 400 nm were chosen for lyophilization process. Other formulations with particle size more than 400 nm or aggregates were excluded.

### 2.3. Lyophilization of nanosuspensions

For lyophilization, the prepared nanosuspension was initially placed under a fume hood for one hour to facilitate the evaporation of the organic phase. After 40 minutes, mannitol or trehalose, serving as lyoprotectants, were added to each sample to achieve final concentrations of 2%, 5%, 7.5%, and 10% (w/v). Then, the nanosuspension was stirred for an additional 20 minutes to ensure complete dissolution of the lyoprotectant. Subsequently, the samples were transferred to a deep freezer (Haier, UK) and stored at −80°C for 24 hours. After freezing, the samples were lyophilized using a freeze dryer (LABCONCO, Kansas, USA) following the method described previously [10]. Lyophilization was done according to the following regime: Primary drying for 90 min at a shelf temperature of −40°C, drying for 90 min at a shelf temperature of −30°C, drying for 90 min at a shelf temperature of −20°C, drying for 530 min at a shelf temperature of −10°C, drying for 90 min at a shelf temperature of 0–10°C. Secondary drying was carried out for 660 min at a shelf temperature of 25°C and a vacuum pressure of 50 mTorr. The resulting lyophilized powders were collected for further analysis.

### 2.4. Analysis of particle size and poly dispersity index (PDI)

A Zetasizer (Malvern Instruments, Malvern, UK) was used to assess the particle size for all nanosuspensions formulations before and after lyophilization. The particle size was obtained as an average in nanometers for three measurement runs. All measurements were carried out at 25˚C and analyzed at a scattering angle of 173°. The polydispersity index (PDI) reflecting the particle size distribution of nanoparticles was also reported.

### 2.5. Zeta potential evaluation

Zeta potentials were determined based on the electrophoretic mobility of the sample using Zetasizer (Malvern Instruments, Malvern, UK) at 25°C. Zeta potential measurements were carried out using folded capillary cuvettes (Folded capillary cell DTS1061, Malvern, UK). Air bubbles were removed from the capillary before measurement. The results were recorded as the average of three measurement run values (mV).

### 2.6. Selection of the optimum formulations after lyophilization

Depending on the results collected after lyophilization, particle size and zeta potential determination of the lyophilized formulations, the optimum formulations with acceptable particle size in nanometer were selected to proceed with the subsequent experiments. Other formulations with particle size more than 400 nm or aggregates were excluded.

### 2.7. X-ray diffraction analysis (XRD)

XRD patterns of Atorvastatin calcium, Mannitol, Pluronic F-127, physical mixture (PM) of ATV/mannitol/Pluronic F 127 and a sample of lyophilized nanosuspension were recorded using an X-ray diffractometer (SHIMADZU, Kyoto, Japan). The samples were scanned from 2 to 65 degrees, with a scanning rate of 2°/min beside the Cu- Ka line as the source of radiation [11,12].

### 2.8. Fourier transform Infrared spectrum of absorption (FTIR)

FTIR spectral analysis of Atorvastatin calcium, Mannitol, Pluronic F-127, PM, and an ATV lyophilized nanosuspension sample was carried out to investigate the changes in the chemical composition of the drug after combining it with excipients. FTIR analysis was carried out using FTIR (Rayleigh, North Carolina, USA). Each sample was dispersed in KBr powder, blended well in a mortar and pestle, and compressed into a transparent disc for examination in the frequency range of 400−4000 cm-1.

### 2.9. Differential scanning calorimetry (DSC) thermal analysis

The DSC experiments have been conducted under a nitrogen environment with a 20 ml/min flow rate using the DSC instrument (SHIMADZU, Kyoto, Japan). DSC was performed for Atorvastatin calcium, mannitol, pluronic F-127, PM and an ATV lyophilized nanosuspension sample. For each DSC run, the aluminum pans containing 2–5 mg of powder material was used. The instrument software program was used to carry out all numerical analysis. The samples were examined at a temperature range of 25°C –250°C with a heating rate of 10°C/min [13].

### 2.10. UV spectroscopy

Specord 50 plus (Analytikjena, Germany) UV-Vis spectroscopy was employed to analyze the absorbance patterns and obtain the UV spectrum for atorvastatin calcium, mannitol, Pluronic F127, trehalose, Cremophor, PVA, PVP K30, and Tween 80. Aqueous solutions of each component were prepared at concentrations corresponding to the expected maximum concentration during the *in vitro* release study. All measurements were scanned in a range between 190–500 nm.

### 2.11. In vitro dissolution test (release study)

The dissolution studies of the optimum lyophilized nanosuspension and the innovator drug Lipitor® were performed using the basket method (apparatus I) Pharma Test dissolution apparatus model PTW II (Hamburg, Germany) operated at 100 rpm. The release was investigated in two media; the first dissolution medium was 900 ml of 0.05 M phosphate buffer pH 6.8. The second dissolution medium was 900 ml 0.1N HCL. Both media were maintained at 37°C ± 0.5°C. Lyophilized samples containing equivalent of 20 mg of atorvastatin calcium were filled into a double zero capsule. Aliquots of 10 ml were withdrawn at different time intervals of 5, 10, 15, 20, 30, and 45 minutes. The withdrawn samples were filtered through a 0.45 μm membrane filter and then analyzed by UV-vis spectroscopy for atorvastatin calcium content at 240 nm. The dissolution experiments were conducted for three samples of each, and the mean values of cumulative drug dissolved were used to plot the release curve.

### 2.12. Statistical analysis

Dissolution data obtained from the lyophilized atorvastatin nanosuspension formulations and the commercial Lipitor® tablets were statistically analyzed using IBM SPSS Statistics software, version 23 (IBM Corp., Armonk, NY, USA). All dissolution experiments were conducted in triplicate, and the results were expressed as mean ± standard deviation (SD). Paired Samples Test analysis was applied to compare the mean dissolution percentages among different formulations at each time point, and P-Value < 0.05 was considered significant.

## 3. Results

### 3.1. Characterization of nanosuspensions

After preparation of the atorvastatin calcium nanosuspensions, they were characterized for the average particle size analysis, PDI, and zeta potential, in which each trial consisted of three runs (n = 3). Any measure above 3000 nm was considered aggregate since the instrument could not measure values above this number. The nanosuspensions with a particle size range between 50–400 nm were chosen and lyophilized and further characterized for the average particle size, PDI, and zeta potential after reconstitution. Each analysis consists of three runs (n = 3).

According to the results after lyophilization, the optimum formulations were selected for further analysis and characterization. Table 2 shows the optimum formulations that exhibited acceptable particle size and PDI. Among these optimum formulations, the formulation that comprised atorvastatin calcium with 2% Pluronic F127 and 80 mg of mannitol after a sonication time of 2 min with an average particle size of 54.47 nm was selected as the best optimum formulation for further characterizations.

**Table 2:** The optimum formulations of atorvastatin calcium after lyophilization using trehalose or mannitol as a lyoprotectant.

Formulation before lyophilization (Stabilizer concentration, sonication time)	Mean particle size ± SD	PDI ± SD	Zeta potential ZP ± SD	Formulation after lyophilization Lyo. (lyophilized)	Mean particle size ± SD	PDI ± SD	Zeta potential ZP ± SD
Atorvastatin- 2% Pluronic F127- 2 min	264.15 ± 52.4	0.836 ± 0.2	0.664 ± 1.1	Lyo. 80 mg mannitol- Atorvastatin- 2% Pluronic F127- 2 min	54.5 ± 0.61	0.141 ± 0.021	−0.809 ± 0.151
Atorvastatin- 1%Tween 80–2 min	527.15 ± 114.7	0.597 ± 0.052	−1.074 ± 0.669	Lyo. 300 mg mannitol- Atorvastatin- 1% Tween 80–2 min	174.4 ± 4.86	0.41 ± 0.047	−0.708 ± 0.64
Atorvastatin- 1% Tween 80–2 min	527.15 ± 114.7	0.597 ± 0.052	−1.074 ± 0.669	Lyo. 80 mg trehalose – Atorvastatin- 1% Tween 80–2 min	138.6 ± 25.9	0.798 ± 0.148	0.25 ± 1.013
Atorvastatin- 2% PVP K30-3 min	202.5 ± 99.8	0.288 ± 0.078	−0.004 ± 0.724	Lyo. 400 mg trehalose- Atorvastatin- 2% PVP K30- 3 min	264.6 ± 14.21	0.466 ± 0.028	0.640 ± 0.692
Atorvastatin- 2% PVP K30-3 min	202.5 ± 99.8	0.288 ± 0.078	−0.004 ± 0.724	Lyo. 400 mg mannitol- Atorvastatin- 2% PVP K30- 3 min	Aggregates		−0.159 ± 1.336
Atorvastatin- 1% HPC- 2 min	260 ± 32.4	0.756 ± 0.055	0.502 ± 0.517	Lyo. 80 mg trehalose- Atorvastatin- 1% HPC- 2 min	380.6 ± 226.8	0.515 ± 0.135	−9.317 ± 1.715
Atorvastatin- 2% PVA- 2 min	539.3 ± 295	0.561 ± 0.211	−0.129 ± 1.35	Lyo. 80 mg trehalose- Atorvastatin- 2% PVA- 2 min	Aggregates		0.009 ± 0.494
Atorvastatin- 0.5% cremophor- 3 min	99.59 ± 88.9	0.507 ± 0.157	−0.369 ± 0.646	Lyo. 400 mg trehalose – Atorvastatin- 0.5% Cremophor- 3 min	70.75 ± 33.3	0.478 ± 0.188	−0.207 ± 0.427
Atorvastatin- 0.5% cremophor- 3 min	99.59 ± 88.9	0.507 ± 0.157	−0.369 ± 0.646	Lyo. 400 mg mannitol- Atorvastatin- 0.5% Cremophor- 3 min	2096 ± 672.6	0.969 ± 0.031	0.123 ± 0.714
Atorvastatin- 1% cremophor- 2 min	378.3 ± 78.5	0.439 ± 0.051		Lyo. 300 mg mannitol- Atorvastatin- 1% Cremophor- 2 min	232.6 ± 4.01	0.527 ± 0.118	−0.23 ± 0.887
Atorvastatin- 1% cremophor- 2 min	378.3 ± 78.5	0.439 ± 0.051	0.383 ± 1.575	Lyo. 300 mg trehalose – Atorvastatin- 1% Cremophor- 2 min	215.7 ± 88.2	0.773 ± 0.219	−0.123 ± 1.443

### 3.2. Poly disparity index (PDI)

PDI determines the particle size distribution on a scale of 0–1. The sample is considered monodispersed when the PDI value is close to zero. PDI values less than 0.1 indicate a very narrow size distribution while values between 0.1 and 0.4 suggest moderate uniformity. On the other hand, values above 0.7 indicate a broad size distribution, giving a significant heterogeneity [14]. The Formulation 2% Pluronic F127, 80 mg mannitol with a PDI value of 0.14 was selected the best optimum formulation showed a uniform sample with a narrow particle size distribution among the prepared optimum nanosuspensions formulations.

### 3.3. Zeta potential values

High absolute zeta potential values (greater than ± 30 mV) generally indicate good stability due to strong repulsive forces preventing aggregation [15]. The low zeta potential of the best optimum formulation was −0.38 ± 0.2 mV, indicating a neutral charge. The used stabilizer (2% Pluronic F127) obviously contributes to stability through the steric hindrance mechanism not due to high zeta potential. In nanosuspensions stabilized through steric mechanism, a low zeta potential does not always indicate poor physical stability [16].

### 3.4. Powder X-ray diffraction (PXRD)

**Fig 2:**
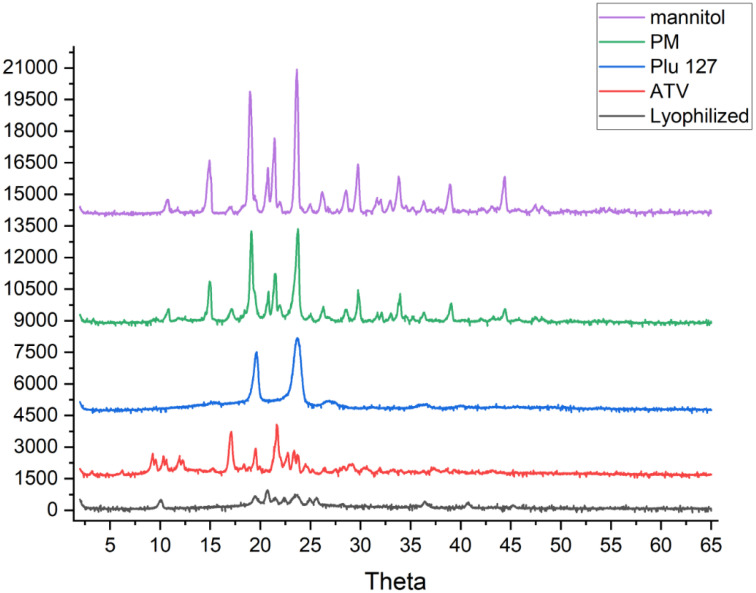
XRD patterns of atorvastatin calcium (ATV), Pluronic F127, mannitol, PM, and a sample of lyophilized nanosuspension.

The PXRD diffraction patterns of atorvastatin calcium, lyophilized sample, physical mixture (PM), mannitol and Pluronic F127 are shown in Fig 2. Atorvastatin calcium being a crystalline material, exhibited sharp diffraction peaks within the 2Theta range of 2–65°, specifically at 10.2°, 17°, 19.4°, 21.5°, and 23.3°. Pluronic F127 is a crystalline polymer exhibiting two sharp peaks at 19.12° and 23.27° in its XRD pattern. Mannitol’s characteristic peaks were sharp and observed at 14.6°, 18.75°, 21.06°, 23.39°, 29.48°, 33.57°, 38.72° and 44.4°. The XRD pattern for the physical mixture (PM) revealed the presence of characteristic peaks corresponding to atorvastatin calcium, Pluronic F127, and mannitol, suggesting that the crystalline structures of the drug, the polymer and the lyoprotectant were preserved within the mixture. In contrast, lyophilized nanosuspension sample exhibited less sharp, intensity and diffuse peaks (weak peaks), with the absence of crystalline sharp peaks displayed in the physical mixture which indicated that the lyophilized nanosuspension assumed a partial conversion to an amorphous state. The reduction in particle size increases the surface area-to-volume ratio, enhancing molecular mobility and promoting amorphization during water removal. It has been known that transforming the physical state of the drug to the amorphous would lead to a high-energy state and high disorder, resulting in enhanced dissolution rate and bioavailability [11].

### 3.5. Infrared spectrum of absorption FTIR

**Fig 3:**
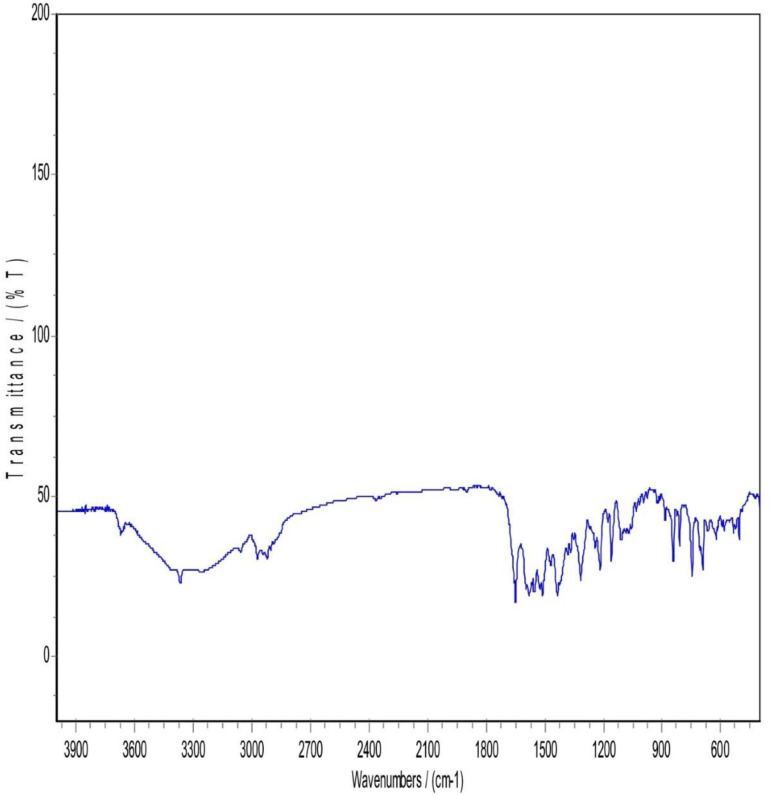
FTIR Patterns of Atorvastatin Calcium.

**Fig 4:**
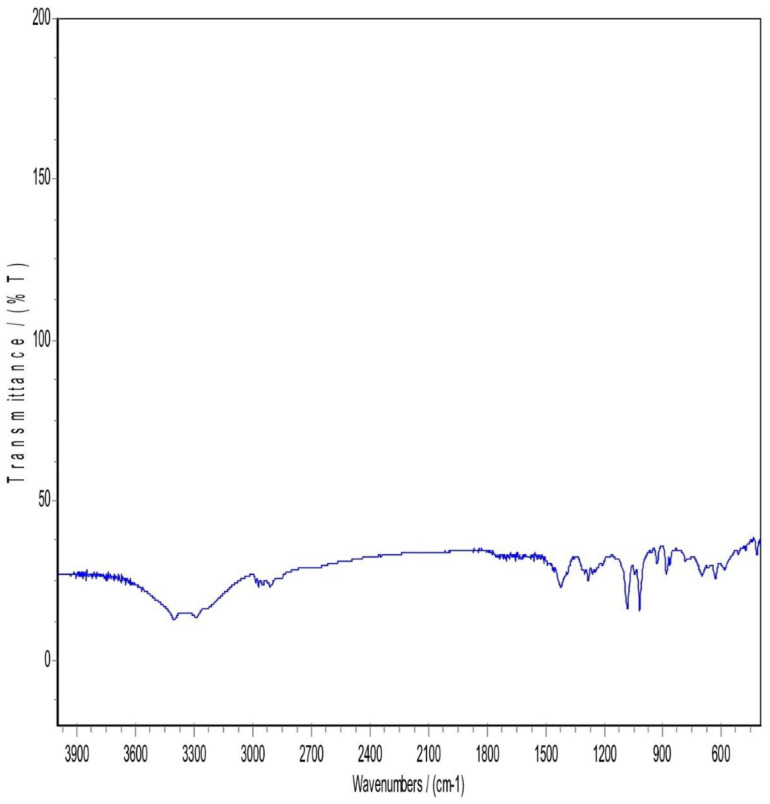
FTIR Patterns of mannitol.

**Fig 5:**
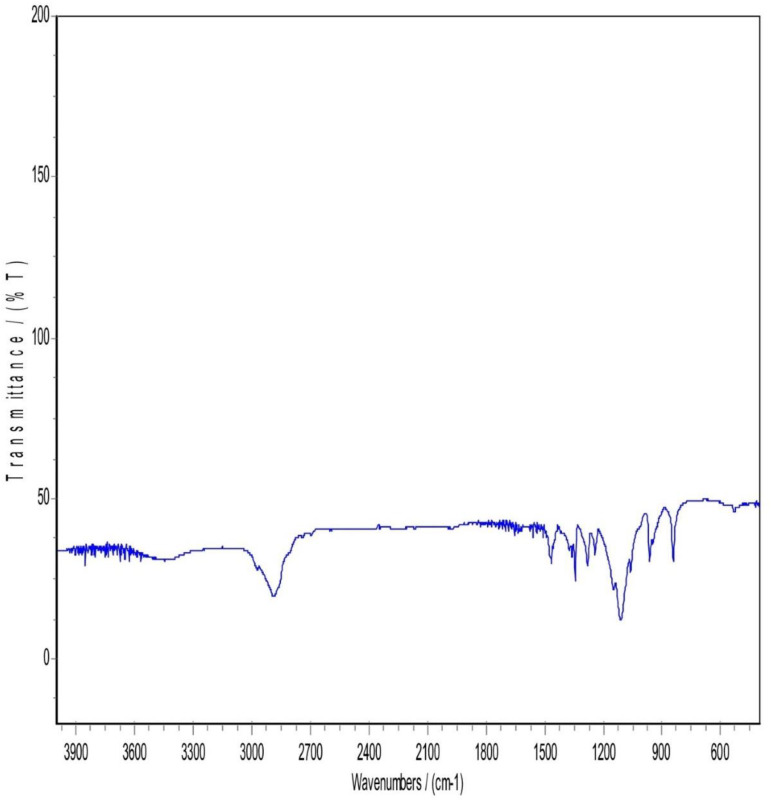
FTIR patterns of Pluronic F127.

**Fig 6:**
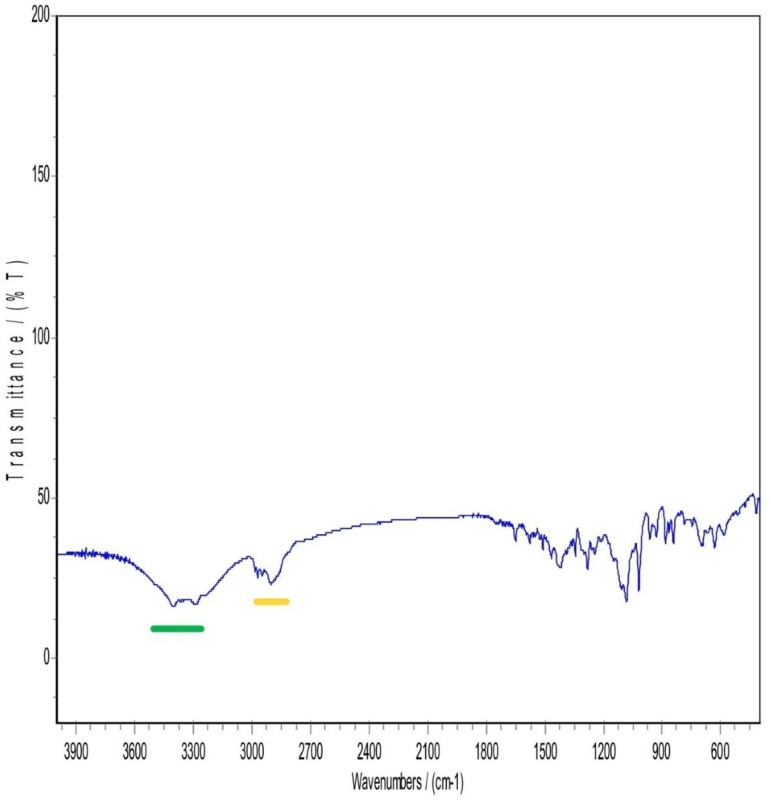
FTIR patterns of Physical mixture (PM).

**Fig 7:**
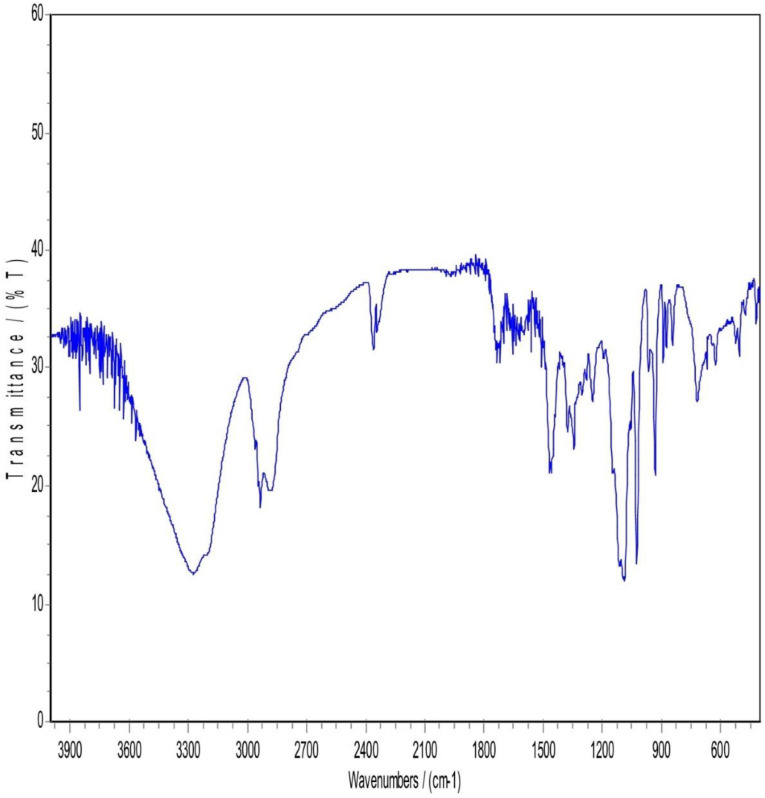
FTIR patterns of the Lyophilized nanosuspension.

FTIR patterns of Atorvastatin calcium, mannitol, Pluronic F127, PM, and a sample of lyophilized nanosuspension of atorvastatin calcium are shown in Figs 3–7, respectively. Atorvastatin calcium exhibits a characteristic peak between 3200–3600 cm-1 (O–H stretching), 3666 cm-1 is assigned to the free (O –H), 3360 cm-1 (N–H stretching band). In addition, 2900 cm-1 (C–H stretching), 1650 cm-1 (amide C = O stretching), 1581 cm-1 (aromatic stretching), 1510 cm-1 (N–H bending), and (C-N) at 1100 cm-1 as shown in Fig 3.

Mannitol FTIR spectrum showed two obvious (O-H) stretches between 3200 cm-1 to 3400 cm-1 and a (C-H stretching) between 2975–2903 cm-1. (C-H scissoring) at 1423.53 cm-1 and (C-H rocking) at 1287.54 cm-1. Moreover, a (C-O stretch) in the range of 1130–960 cm-1 (Fig 4). Pluronic F-127 has two main characteristic peaks, a (C-H stretching) at 2889 cm-1 and a (C-O stretch) in the range of 1130–960 cm-1 (Fig 5).

In Fig 6, The FTIR Spectrum of the physical mixture (PM) displayed notable characteristics, particularly in the region between 3300–3670 cm-1. This region revealed either concealment or loss of the drug characteristic peak, possibly due to the influence of mannitol and Pluronic F127. Both Pluronic F127 and mannitol kept nearly the same spectral features. For atorvastatin calcium, a complete disappearance of O-H stretching peaks between 3200–3666 cm-1 and N-H stretching peak that appeared approximately around 3360 cm-1. This could be attributed to the potential dilution of the drug within the physical mix, given the drug’s lower proportion in the mixture (drug: mannitol: polymer ratio of 1: 4: 4). Alternatively, it could be explained by the possibility of hydrogen bonding between the O-H and N-H groups of atorvastatin calcium with (O-H) in mannitol or bonding to (NH) with another neighboring OH group coming from mannitol. Mannitol and Pluronic F127 may formed hydrogen bonds with Atorvastatin calcium. These hydrogen bonds could alter the vibrational frequencies of N-H and O-H bonds, causing shifts or broadening of the peaks and in some cases, their disappearance from the spectrum. Considering the above explanation, a suppression of the carbonyl group peak with a very slight shift was observed in the spectral region between 1500−1800 cm-1.

Regarding Fig 7, Lyophilization is a freezing and drying process that can result in production of a more amorphous state in the sample. This state can alter the vibrational modes of functional groups, potentially leading to differences in peak positions and intensities when compared to the drug or other components in the sample to the lyophilized form. So, it was noticed that in the region between 3000–3700 cm-1, a complete disappearance of atorvastatin calcium (N-H) and (O-H) peaks, while a full appearance of broad (O-H) stretching peak. This may be explained by the fact that the polymer and lyoprotectant successfully preserved and coated the drug nanoparticles. An increased peak intensity of C = O bond stretching at 2400 cm-1 was observed in the lyophilized sample while not observed in the physical mixture sample. It is anticipated that it was due to the presence and interaction of mannitol and pluronic F127 in the nanosuspension. Unlike the physical mixture, the nanosizing process might enhance certain regions of the spectrum. Lyophilization can induce amorphization as said before, and this change in the physical state may lead to different vibrational modes in the FTIR spectrum. Accordingly, a shift and suppression of peaks below 1800 cm-1 in the FTIR spectrum of the lyophilized nanosuspension were observed. In addition, a strong absorption at 1130–960 cm-1 appeared in the lyophilized sample and that could be attributed to the existence of Pluronic F127 and mannitol when both have strong absorption in this band. The most characteristic peaks of atorvastatin calcium were still present but some decrease in the intensity and shifting of some peaks and this indicated that there was no chemical incompatibility between the drug and the carrier [17].

### 3.6. Differential scanning calorimetry DSC

**Fig 8:**
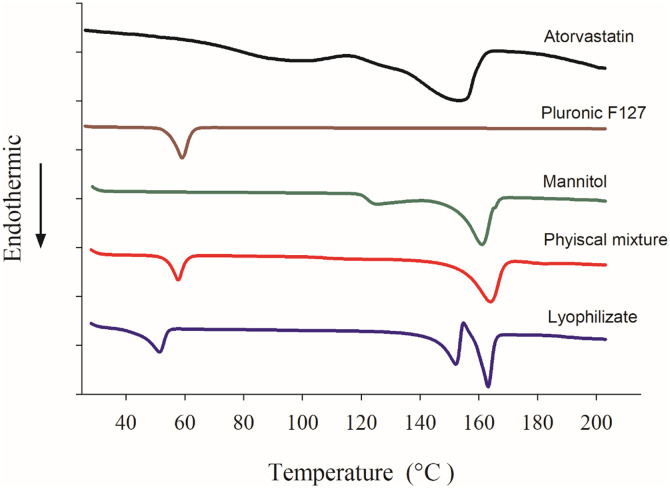
DSC thermograms of atorvastatin calcium (ATV), mannitol, pluronic F127, physical mixture, and a lyophilized nanosuspension sample.

DSC was used to study the thermal behavior and the solid state after lyophilization, and further compare it with other materials used in the preparation. Also, it was used to verify the successful coating of atorvastatin calcium nanocrystals by the stabilizer and the lyoprotectant. The DSC thermograms of atorvastatin calcium, pluronic F127, mannitol, physical mixture (PM), and a lyophilized sample of atorvastatin calcium nanosuspension are shown in Fig 8. Atorvastatin calcium exhibited a sharp endotherm at 153.13°C corresponding to the melting point of the drug. The broad endothermic peak from 68–114°C could be attributed to the loss of water molecules. DSC thermogram of pluronic F127 showed a sharp endothermic peak at 58.99°C corresponding to its melting point. DSC thermogram of mannitol revealed an endothermic peak at 161.3°C corresponding to its melting point. The physical mixture PM DSC thermogram showed two peaks; one of them referred to pluronic F127 at 57.75°C. The second peak was at 163.97 °C and it most likely corresponded to mannitol which was present at 4:1 ratio to atorvastatin. Another possible explanation for the absence of an atorvastatin peak was that it was shifted to a higher value and appeared as one peak with mannitol at 163.97 °C due to the interaction with mannitol in the molten pluronic F127. Finally, lyophilized nanosuspension displayed three endothermic peaks; one at 51.41°C corresponding to pluronic F127, and two peaks at 152.09°C and 163.21°C representing atorvastatin calcium and mannitol, respectively. For atorvastatin calcium peak at 152.09°C, there appeared a slight shift of the endotherm from 153.13°C reported above of atorvastatin alone. This indicated that some of the crystalline nature of atorvastatin has been preserved after nanosization and lyophilization.

### 3.7. UV spectroscopy analysis

**Fig 9:**
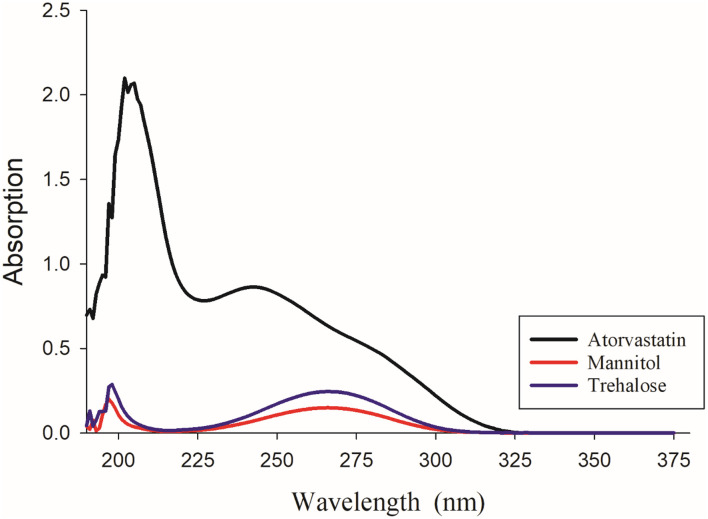
UV Spectrums of atorvastatin calcium, mannitol, and trehalose.

**Fig 10:**
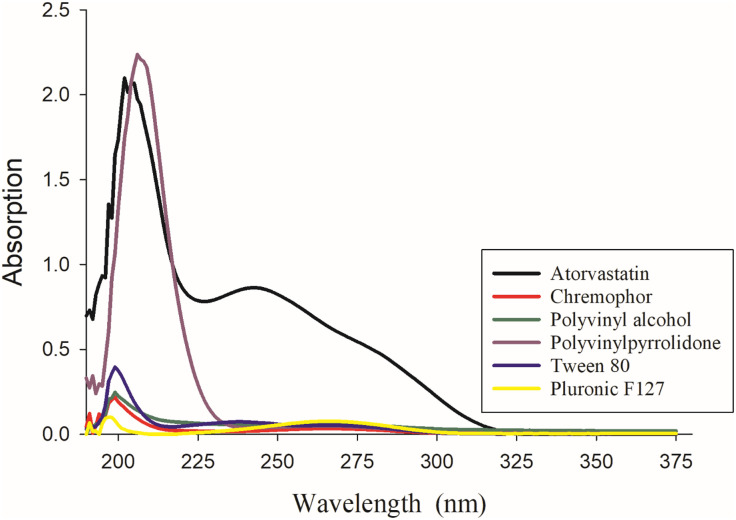
UV Spectrums of atorvastatin calcium and the stabilizers for optimum formulations; Chremophor, PVA, PVP K30, Tween 80 and pluronic F127.

**Fig 11:**
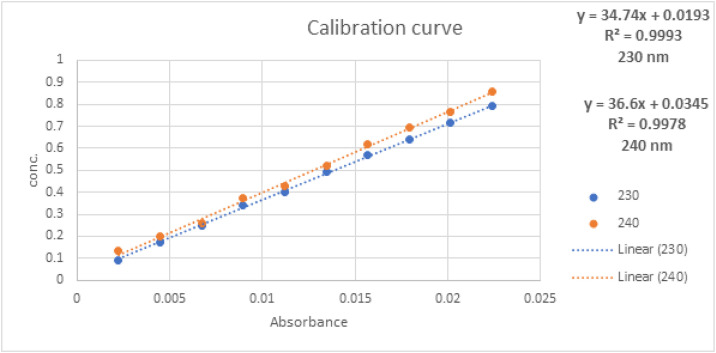
Linearity calibration curve of atorvastatin calcium at 240 nm and 230 nm.

The UV spectra for various materials used in this study are presented in Figs 9 and 10. Atorvastatin calcium exhibited a UV absorption maximum at 244 nm. Both pluronic F127 and mannitol displayed absorption maxima at a slightly longer wavelength of 264 nm. It appeared that the absorption from various additives had minimum interference with the model drug in the range of 230–240 nm. Accordingly, two calibration curves were constructed at 230 nm and 240 nm (Fig 11). The linearity of the calibration curves for atorvastatin calcium was calculated and constructed by the least square regression method. The correlation coefficients (R2) for the standard calibration curves at 230 nm and 240 nm for atorvastatin calcium were 0.9993 and 0.9978, respectively.

### 3.8. In vitro dissolution study

**Fig 12:**
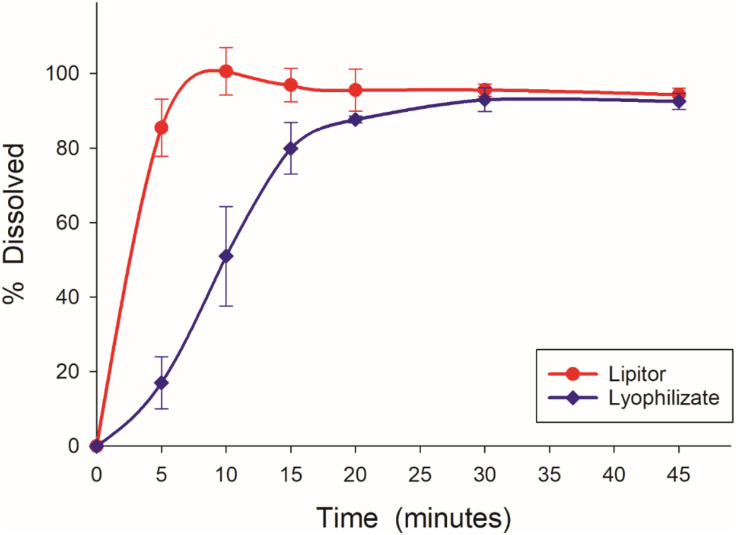
Dissolution profile of Lyophilized sample and the original drug Lipitor® in 0.05 M potassium phosphate buffer.

**Fig 13:**
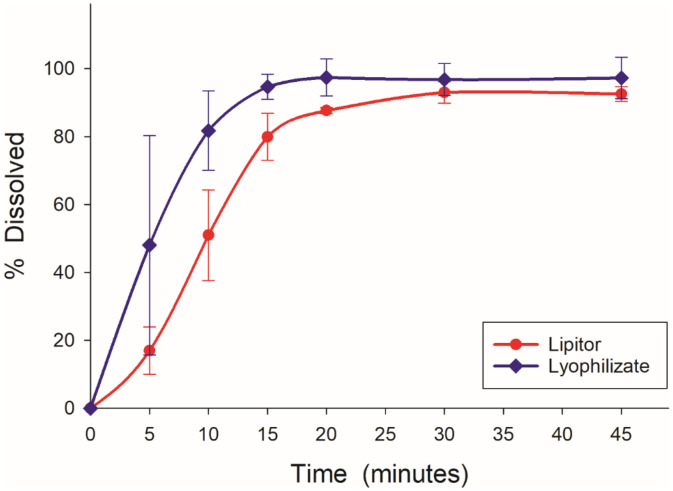
Dissolution profile of lyophilized sample and the original drug Lipitor® in 0.1N HCL.

Lyophilized nanosuspension exhibited a slower dissolution characteristic than the original drug Lipitor® in the compendial medium (phosphate buffer pH 6.8) as shown in Fig 12. The innovator exhibited a rapid initial release of around 80% after 5 minutes and a complete release after 10 minutes. On the other hand, the lyophilized sample demonstrated a slower initial release around 20% after 5 minutes but continued to dissolve steadily exceeding 80% release after 20 minutes. In contrast, the dissolution profile in 0.1N HCL showed that the lyophilized nanosuspension exhibited faster release than the innovator. After 5 minutes for example, the lyophilized nanosuspenstion released 49% of the model drug as compared to 18% from Lipitor® (Fig 13).

The mean value for Lipitor® (0.851 ± 0.045) was higher compared to that of the lyophilized sample (0.465 ± 0.240) when tested in 0.05 M PBS. The p-value obtained was 0.007, which is below the 0.05 significance threshold, indicating a statistically significant difference between the two groups. These findings suggested that Lipitor® exhibits superior performance in this medium compared to the lyophilized sample.

The mean value for the lyophilized nanosuspension (0.555 ± 0.157) was higher compared to the innovator product, Lipitor® (0.474 ± 0.106), when tested in 0.01 N HCl. The p-value was 0.029, which was below the 0.05 significance threshold, indicating a statistically significant difference between the two formulations. These results demonstrated that the lyophilized nanosuspension exhibited superior performance in this medium compared to the innovator formulation.

The later result was not surprising as the nanosuspesion formation reduced the particle size and increased the surface area; consequently, improving the dissolution rate. Moreover, nanosization process coupled with lyophilization appeared to result in partial amorphization, which would also contribute to the enhanced dissolution.

## 4. Discussion

The pharmaceutical industry faces many problems with poor aqueous solubility of drugs and thus, their limited bioavailability [1]. Several techniques have been used in literature to enhance the solubility or the dissolution of these drugs [1]. Nanosuspension was shown to be a promising and efficient strategy for enhancing the dissolution of poorly soluble drugs [1]. Atorvastatin calcium, a lipid-lowering drug falls within the BCS class-II category, characterized by low solubility and high permeability. Consequently, any attempt aimed to enhance the solubility of atorvastatin calcium or the dissolution could lead to improved bioavailability. Considering this, the present study aimed to develop nanosuspension of atorvastatin calcium and assessed its properties and *in vitro* release studies.

Among various pharmaceutical approaches, nanoparticle holds significance today [18]. Different methods have been proposed for nanosuspension preparation. Two different methods are employed; top-down methods and bottom-up methods. Top-down methods utilize the disintegration process to transform large particles to microparticles and then to nano-sized particles, while bottom-up method based on assembly process that generates nanoparticles from molecules [19]. Bottom-up methods including supercritical fluid, antisolvent precipitation, emulsification-solvent evaporation, and lipid emulsion/micro-emulsion template methods. Top-down methods including non-aqueous media high-pressure homogenization, (Nanopure) aqueous media high-pressure homogenization (Dissocubes), media milling (Nanocrystals), high-pressure homogenization and precipitation (Nanoedge), and Nano-jet technology [19].

However, anti-solvent nanoprecipitation was selected due to its simplicity at the laboratory scale, and ability to generate small particle sizes [20]. Many factors influence the particle size of nanosuspensions, including the choice of the stabilizer which plays a crucial role in determining the particle size of nanosuspensions. In this study, the atorvastatin calcium nanosuspensions were prepared using different stabilizers at different concentrations such as PVP, cremophor, Pluronics and Tweens and characterized to identify the potential optimum formulations. Different promising formulations were obtained after the lyophilization of formulations that have a size lower than 400 nm. A formulation with particle size that was significantly reduced from 242.4 nm to 54 nm after lyophilization and reconstitution was prepared with 2% pluronic F127 and 80 mg mannitol as a stabilizer and lyoprotectant, respectively. Our finding suggested that beyond the particle size change, some other factors related to the stabilizer used may be predominantly influencing the release of atorvastatin calcium. Overall analysis of atorvastatin calcium nanosuspension suggested the particle size or the solubility of nanosuspension appeared to be dependent on the stabilizer characteristics and its concentration. As demonstrated in the table provided, the use of lyoprotectants could lead to maintain, reduce, or increase the particle size. For example, in the nanosuspension containing 2% pluronic F127, the particle size measures 242 nm. Subsequently, after undergoing lyophilization with a 2% mannitol solution, the particle size diminishes to approximately 54.5 nm. Conversely, the addition of 2% trehalose results in an increase in particle size to 631.4 nm. It is important to note that an increase in lyoprotectant concentration mostly correlates with an increase in particle size. These changes in particle size may be attributed to the nature properties and behavior of lyoprotectants during the freeze-drying process, as well as their adsorption onto the nanoparticle surface [21]. Furthermore, the polydispersity index (PDI) of 0.14 reflected a narrow size distribution, indicative of uniform particle sizes within the nanosuspension. This uniformity was essential for ensuring consistent drug delivery and minimizing variability in therapeutic outcomes. Additionally, the measured zeta potential value of −0.809 mV was low indicated a neutral charge, suggesting that the stability was maintained by the steric hindrance mechanism. The used stabilizer (2% Pluronic F127) obviously contributes to stability through the steric hindrance mechanism not due to high zeta potential. In nanosuspensions stabilized through steric mechanism, a low zeta potential does not always indicate poor physical stability [16]. Hashem *et al*., highlighted the relatively low zeta potential value observed in their study, reporting approximately 7.2 mV for a solid nanosuspension of atorvastatin with Plurnic F127 and with a particle size of 260 nm. This low zeta potential could affect the stability of atorvastatin calcium nanosuspension, potentially impacting its storage and necessitating the need for freshly prepared nanosuspension before use which may affect the storage of the prepared nanosuspension. The authors underscored the critical nature of the environment in which nanosuspensions were spontaneously formed, emphasizing the importance of the technique used in the formulation process [22].

The FTIR spectral analysis revealed several interesting observations. First, there was an increased peak intensity of C = O bond stretching at 2400 cm-1. Second, the absorption at 3666 cm-1 which was assigned to the free OH stretching was observed in the spectrum of atorvastatin calcium, while not observed in the lyophilized nanosuspension. Finally, a sharp peak at 3360 cm-1 that corresponds to NH stretching was found in the FTIR spectrum of atorvastatin calcium, while broadband was observed for lyophilized nanosuspension. These observations suggested that partial amorphization of the drug occurred during the processing of the nanosuspension. Furthermore, it was indicative of an interaction, most probably physical interaction, between the additives and the drug. In other words, the polymer and lyoprotectant successfully preserved and coated the drug nanoparticles.

The crystalline state affects the solubility and dissolution of a compound [23,24]. The crystalline state of the samples was evaluated to prove the effect of nanosization on the physical state of atorvastatin calcium. Atorvastatin calcium, pluronic F127, and mannitol were completely crystalline materials as indicated by their respective thermograms and PXRD scans as well as those of the physical mixture (Figs 2 and 8). On the other hand, Atorvastatin calcium was transferred to a partially amorphous form during the processing of the final lyophilized nanosuspension. The DSC thermograms of the lyophilized formulation illustrated that atorvastatin calcium was partially amorphous in this system. A similar observation was also reported previously [25]. Also, X-ray powder diffraction data showed that lyophilized nanosuspension did not display any crystalline sharp peaks of Atorvastatin calcium. In support to the FTIR and DSC observations, the PXRD data also provided convincing evidence of coating the drug by the polymer and excipients. The dissolution test results of lyophilized nanoparticles and the innovator were analyzed to assess their respective dissolution rates. Lyophilized nanosuspension had superior dissolution in acidic media which highlighted the benefits of nanosization in producing more dissolvable dosage forms, especially under conditions that mimic the gastric environment. The solubility of atorvastatin calcium is much lower in an acidic medium as compared to that at pH 6.8 (Table 1). Specifically, the reported solubility in 0.1 N HCl is 0.0315 mg/ml while it is 0.2838 mg/ml at pH 6.8. Being an immediate-release product that would disintegrate and start to release the drug in the stomach, the prepared nanosuspension could have a potential advantage over the conventional dosage form.

Interestingly however, nanosuspension formulation was inferior to the innovator in terms of release at pH 6.8. In this medium where the drug is more soluble, it would have been expected that both products show similar rapid dissolution. Nevertheless, the innovator completely released the drug in 10 minutes. On the other hand, a complete release was achieved in 20 minutes for the nanosuspension at a slower rate. These findings highlighted the critical role of excipient selection and functionality in the formulation of nanoparticles, particularly the impact of additives on drug release kinetics.

## 5. Conclusion

The main objective of the conducted study was to develop nanosuspensions of atorvastatin calcium using the antisolvent precipitation technique and characterization of the prepared nanosuspensions. This was followed by conversion of the aqueous dispersion into a powder form by lyophilization, and into a solid dosage form (capsules) using suitable additives. The best optimum and promising formulation characterized in this study was 2% pluronic F127 as a stabilizer with 80 mg mannitol as a lyoprotectant. The optimized formulation of the nanosuspension showed a particle size of 242.4 ± 30.4 before lyophilization and a particle size of 54.5 after the lyophilization step. Characterization techniques such as XRD, DSC, and FTIR were employed. Collectively, they revealed a reduction in crystallinity, indicating the conversion of atorvastatin calcium from its crystalline form to partially amorphous form when processed. Also, the polymer and lyoprotectant successfully preserved and coated the drug nanoparticles. This study has also provided an important insight into the dissolution characteristics of the lyophilized nanosuspension compared to the innovator Lipitor® in different dissolution media. The results demonstrated that Lipitor® exhibited a higher percentage dissolved in 0.05 M phosphate buffer, while the lyophilized sample showed superior dissolution in 0.1N HCl. The dissolution study highlighted the significant impact of formulation and processing on the release and the potential bioavailability of atorvastatin calcium. Nanosization alone did not guarantee enhanced dissolution in all bio-relevant media. Excipient functionality should not be overlooked as it may counteract the anticipated outcomes. The results of this study showed that formulation parameters such as stabilizer type and concentration played an important role in nanosuspension preparation methods. Nanosuspension represents a promising alternative to current delivery systems aimed to improving the biopharmaceutical performance of poorly water-soluble drugs.

## Supporting information

S1 File DSC data. (ZIP)

S2 File FTIR data. (ZIP)
