# Supplementary material for: Preparation, characterization and in vitro evaluation of atorvastatin nanosuspensions
Source: PLoS One. 2025 Oct 21;20(10):e0335024. doi: 10.1371/journal.pone.0335024 (PMC12539708; doi:10.1371/journal.pone.0335024)
Supplement: S2 File — (ZIP) [file pone.0335024.s002.zip › FTIR data/lyo- pm.pdf]

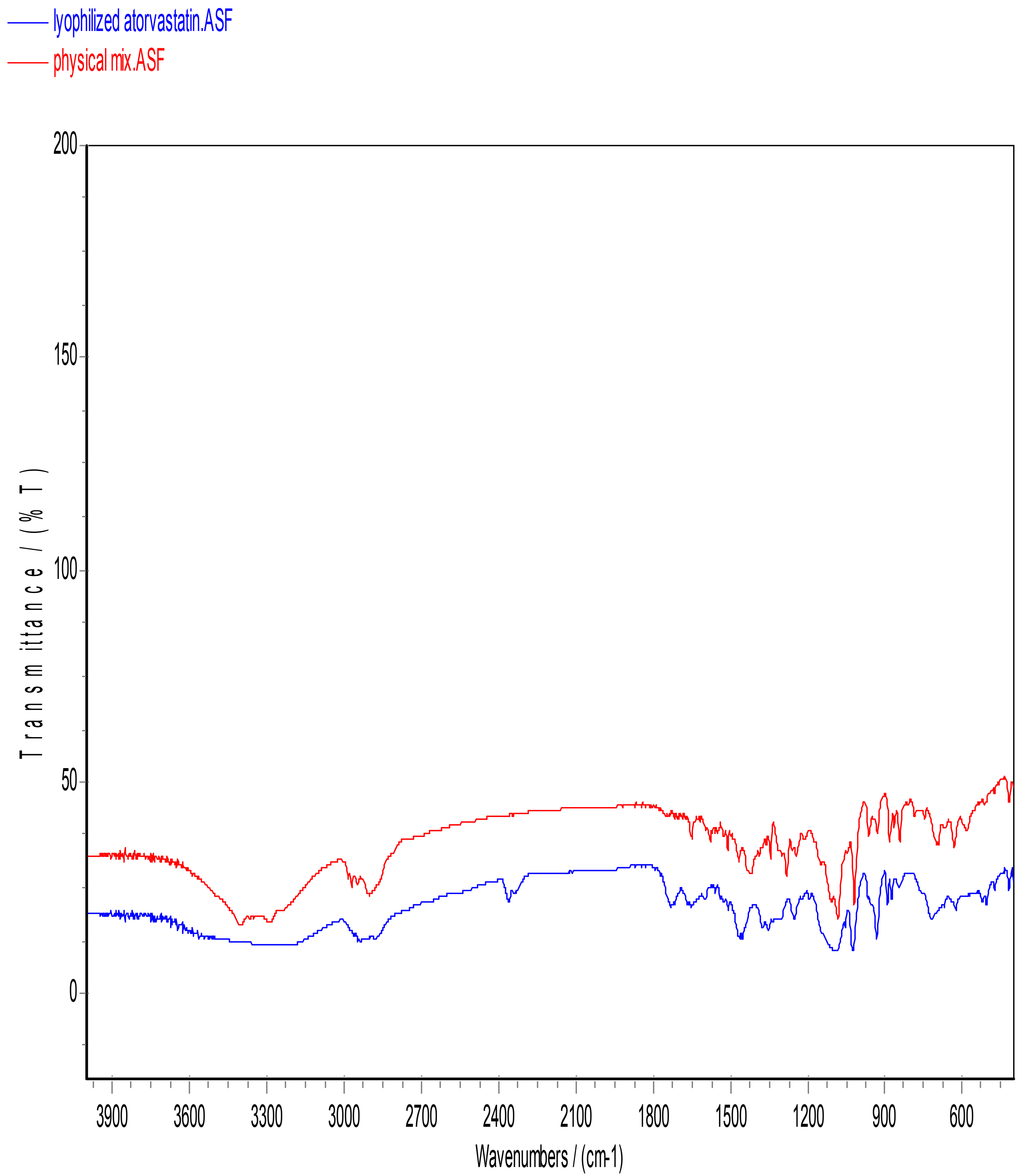

Instrument model=WQF-520 resolution=4 scan times=32

2023/9/18 3:39 physical mix.ASF
