# Supplementary figures and images for: Preparation, characterization and in vitro evaluation of atorvastatin nanosuspensions
Source: PLoS One. 2025 Oct 21;20(10):e0335024. doi: 10.1371/journal.pone.0335024 (PMC12539708; doi:10.1371/journal.pone.0335024)

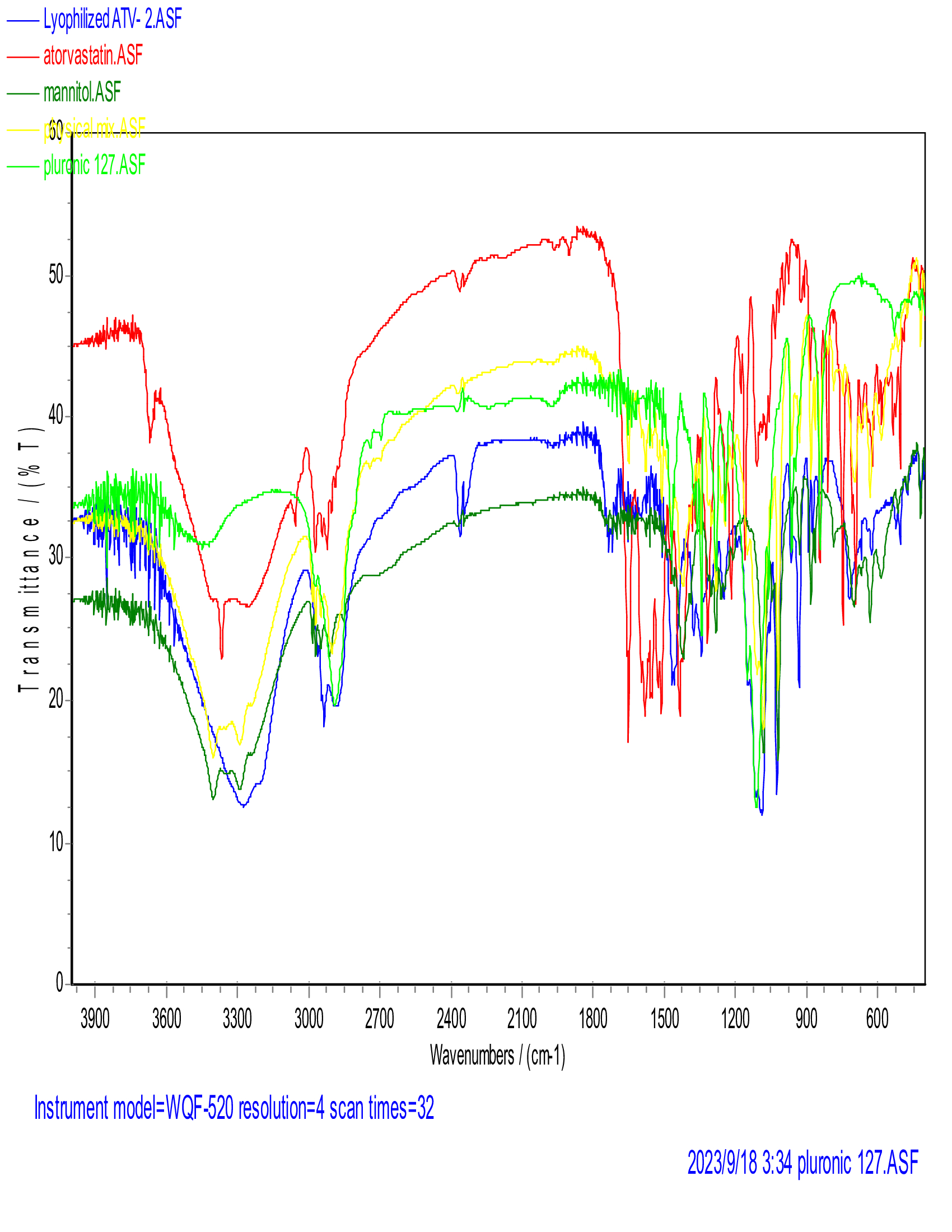

Supplement: S2 File — (ZIP) [file pone.0335024.s002.zip › FTIR data/all.jpg]

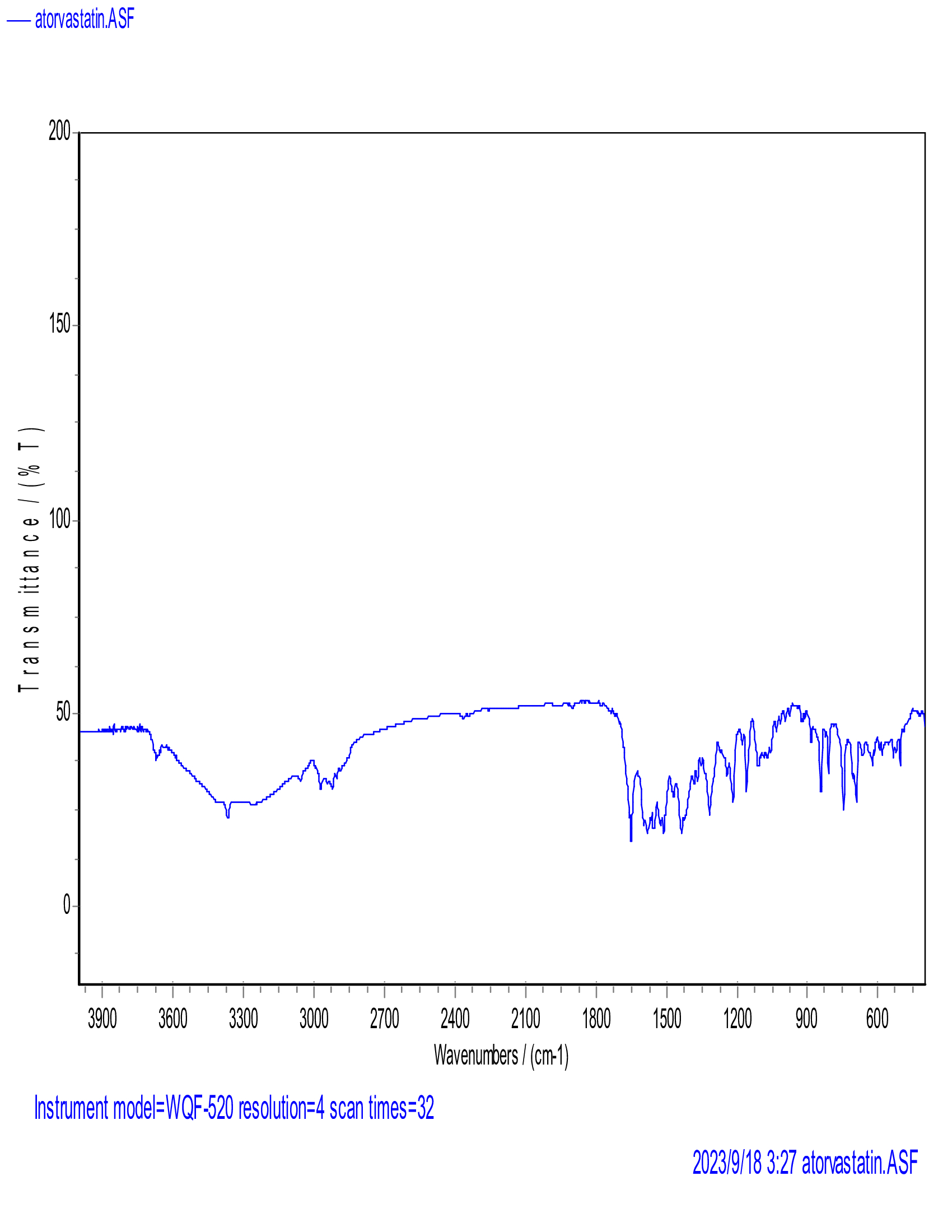

Supplement: S2 File — (ZIP) [file pone.0335024.s002.zip › FTIR data/atorvastatin.jpg]

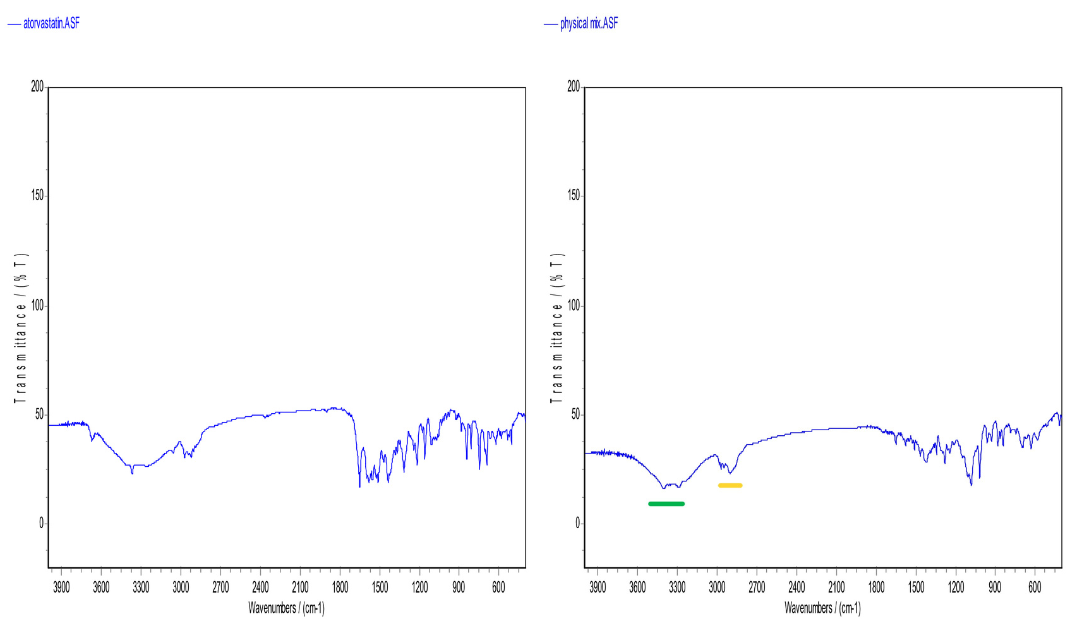

Supplement: S2 File — (ZIP) [file pone.0335024.s002.zip › FTIR data/ATV- PM.png]

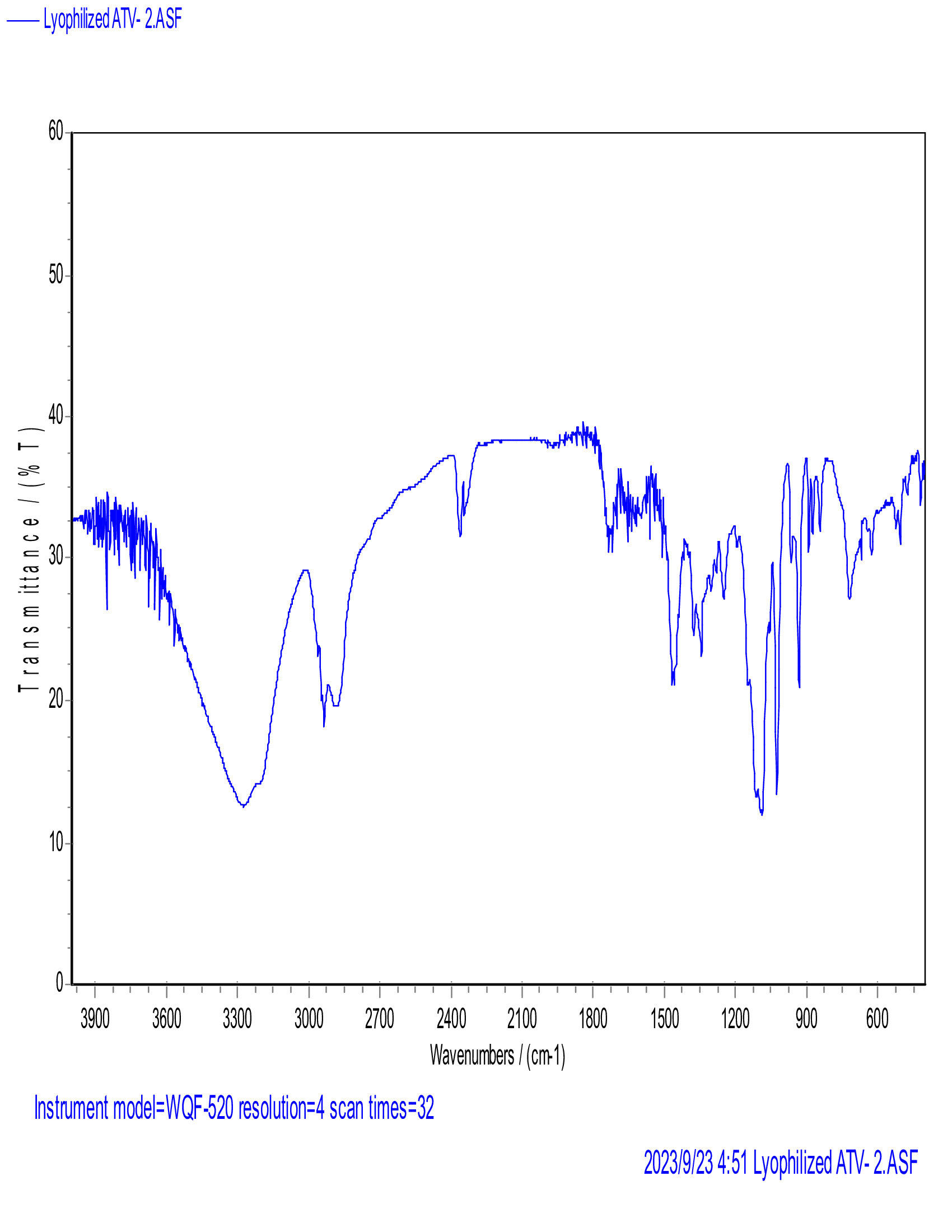

Supplement: S2 File — (ZIP) [file pone.0335024.s002.zip › FTIR data/lyo.jpg]

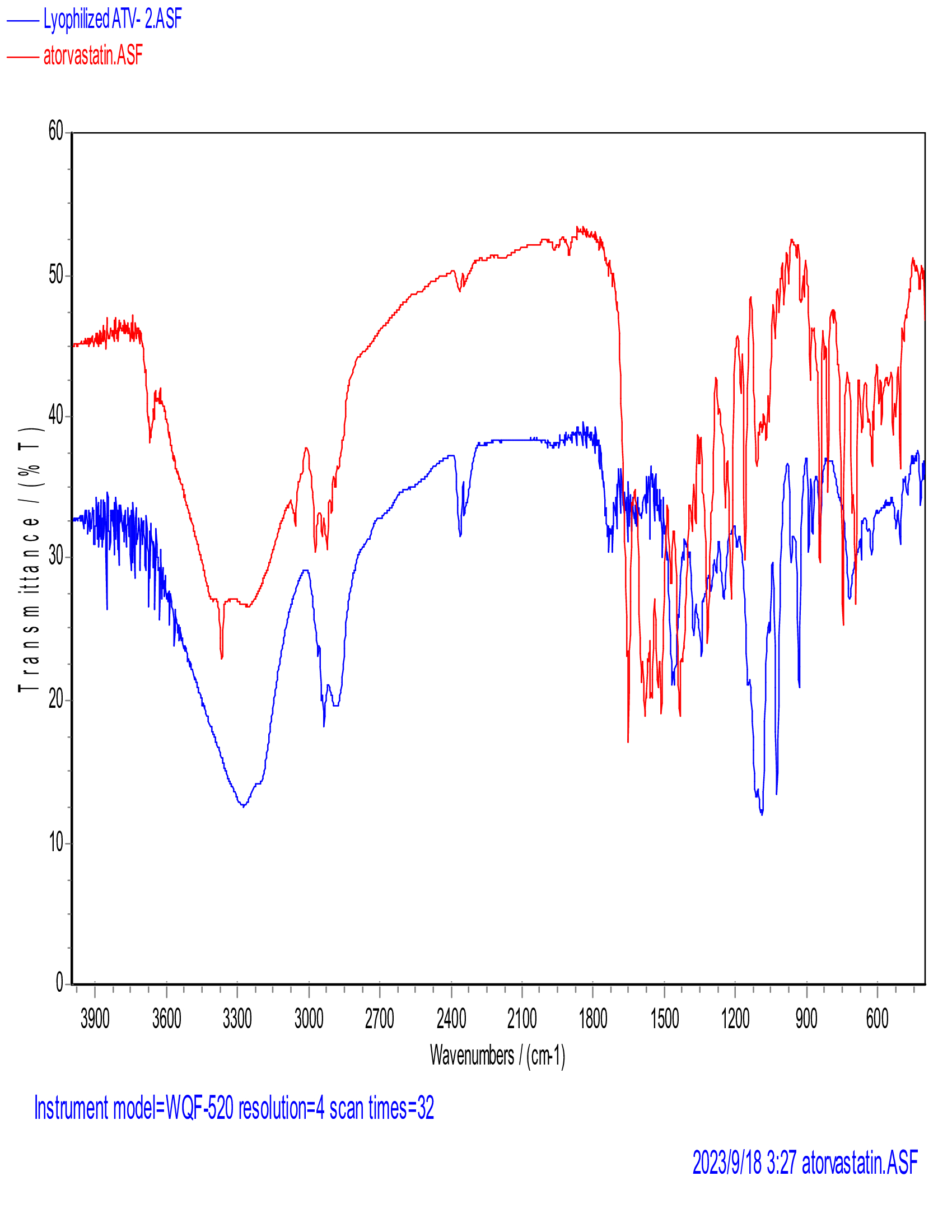

Supplement: S2 File — (ZIP) [file pone.0335024.s002.zip › FTIR data/lyo2-ATV(1).jpg]

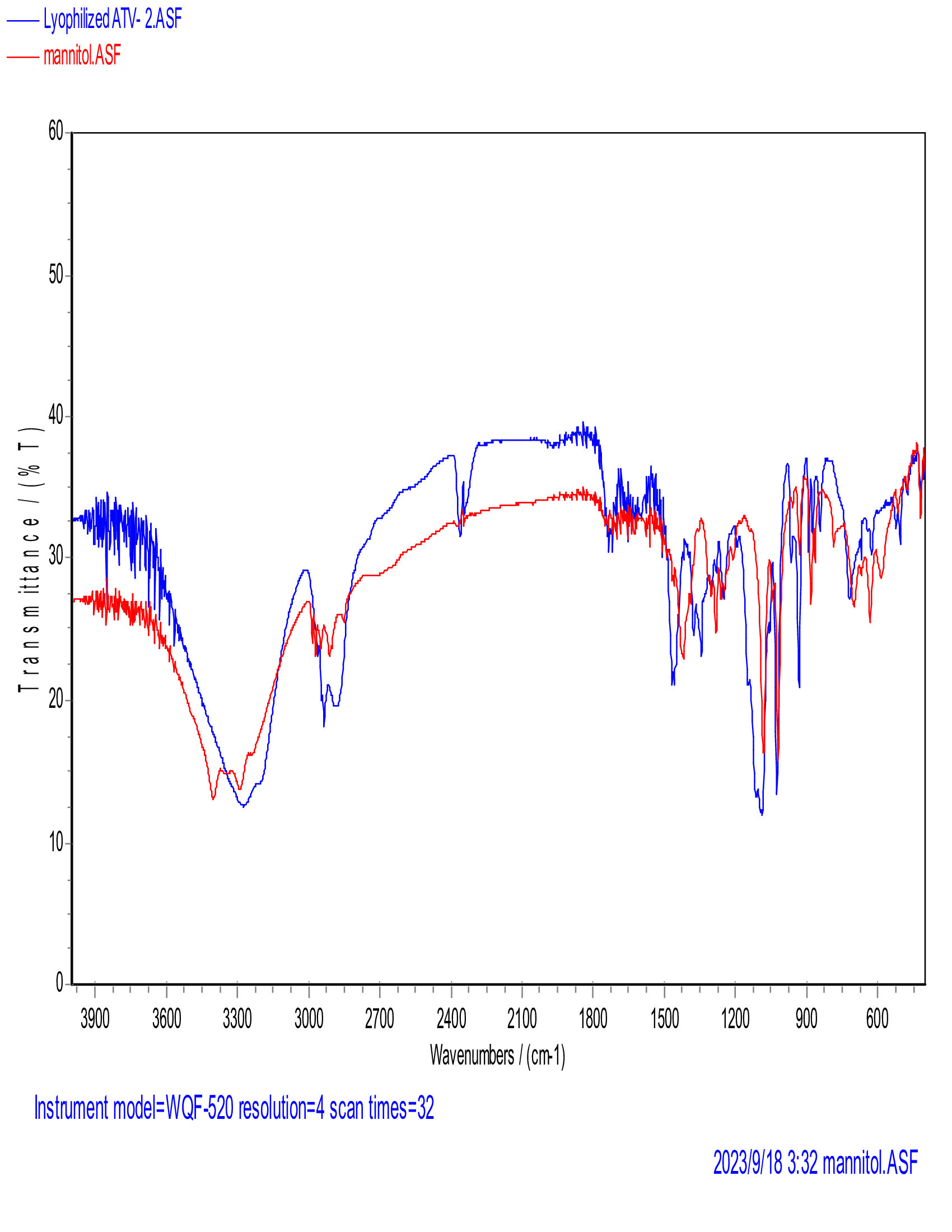

Supplement: S2 File — (ZIP) [file pone.0335024.s002.zip › FTIR data/lyo-mannitol.jpg]

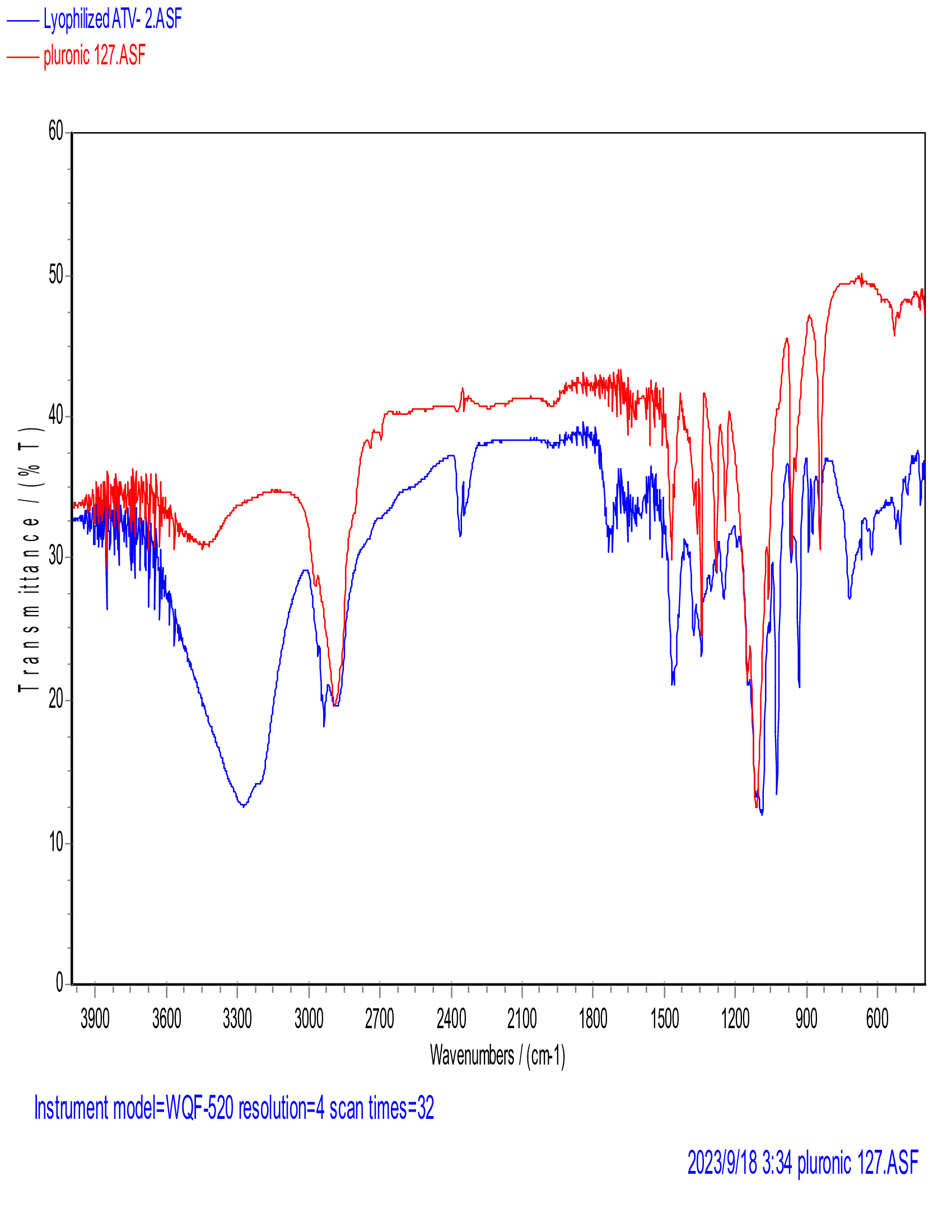

Supplement: S2 File — (ZIP) [file pone.0335024.s002.zip › FTIR data/lyo-pluronic.jpg]

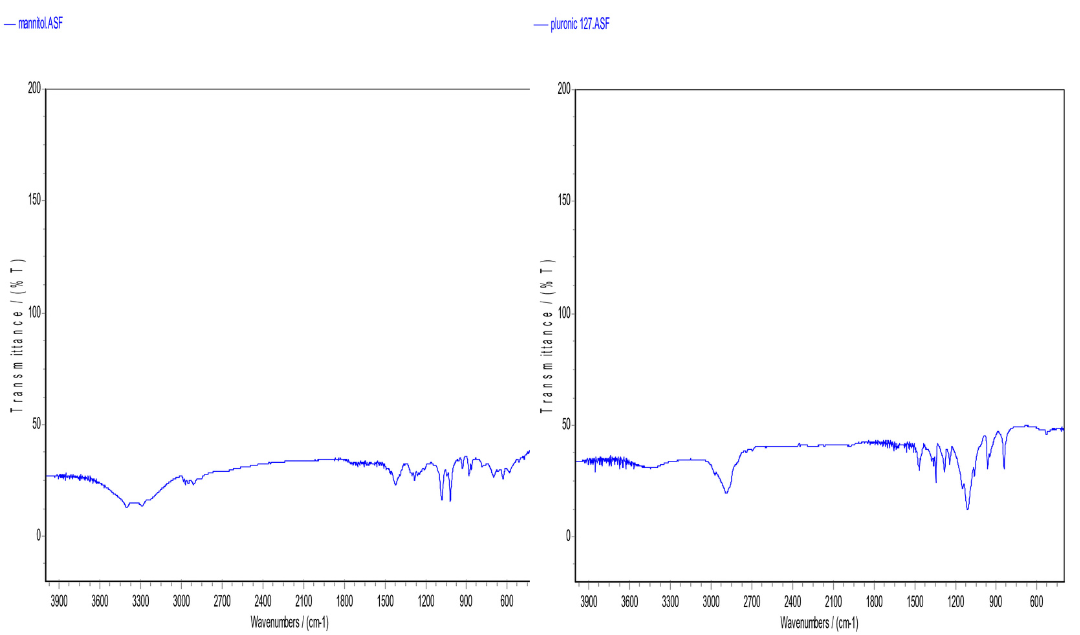

Supplement: S2 File — (ZIP) [file pone.0335024.s002.zip › FTIR data/mann- plu.png]

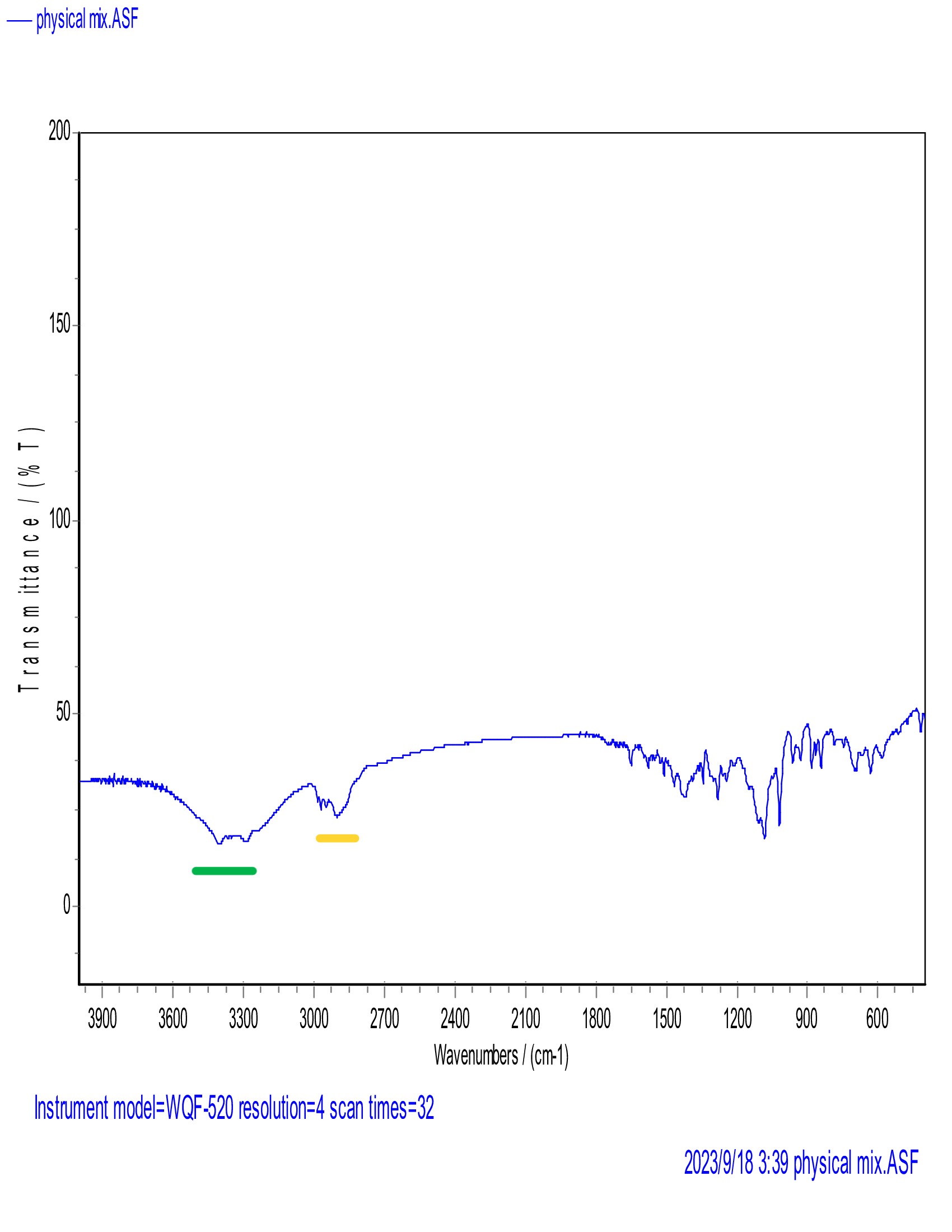

Supplement: S2 File — (ZIP) [file pone.0335024.s002.zip › FTIR data/physical-mix.jpg]

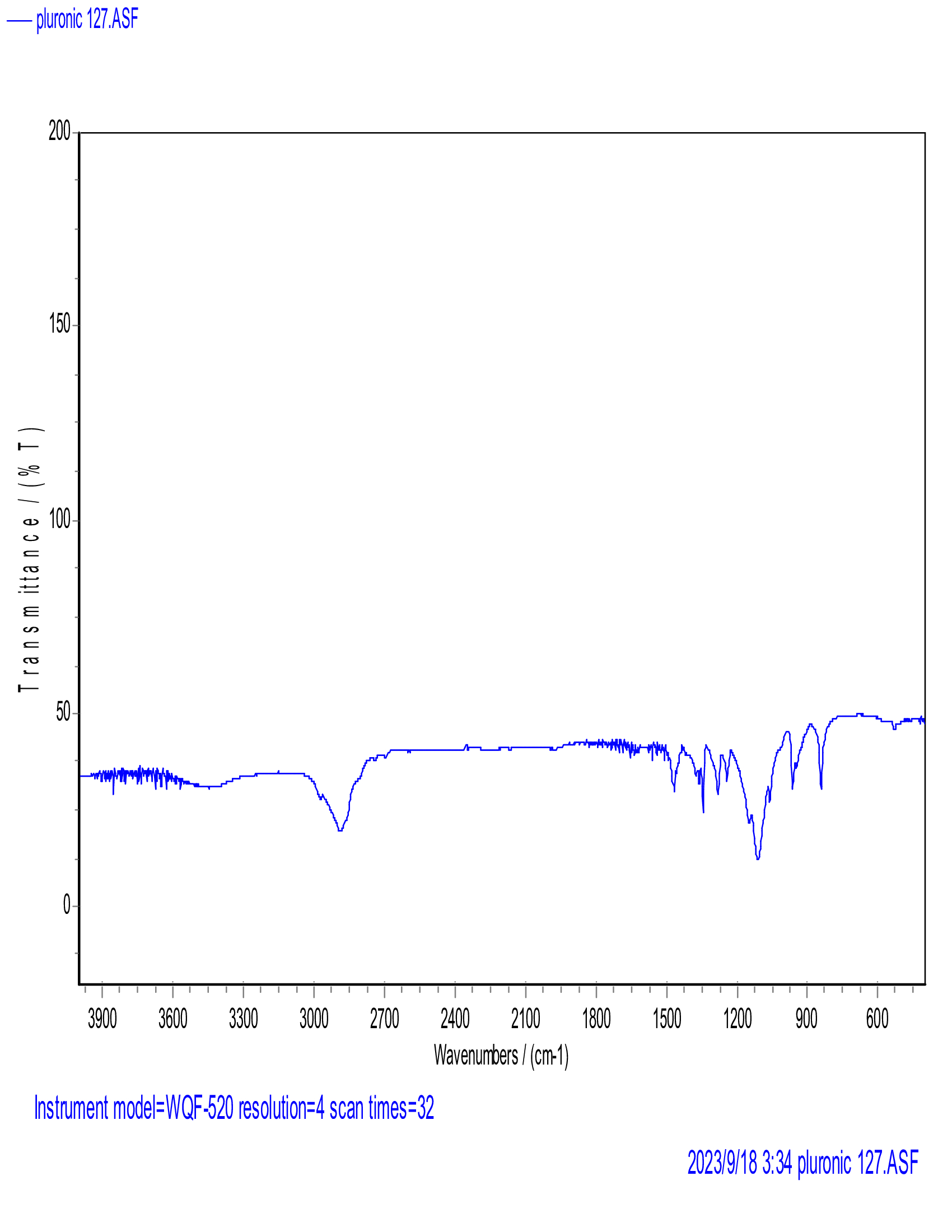

Supplement: S2 File — (ZIP) [file pone.0335024.s002.zip › FTIR data/pluronic-127.jpg]

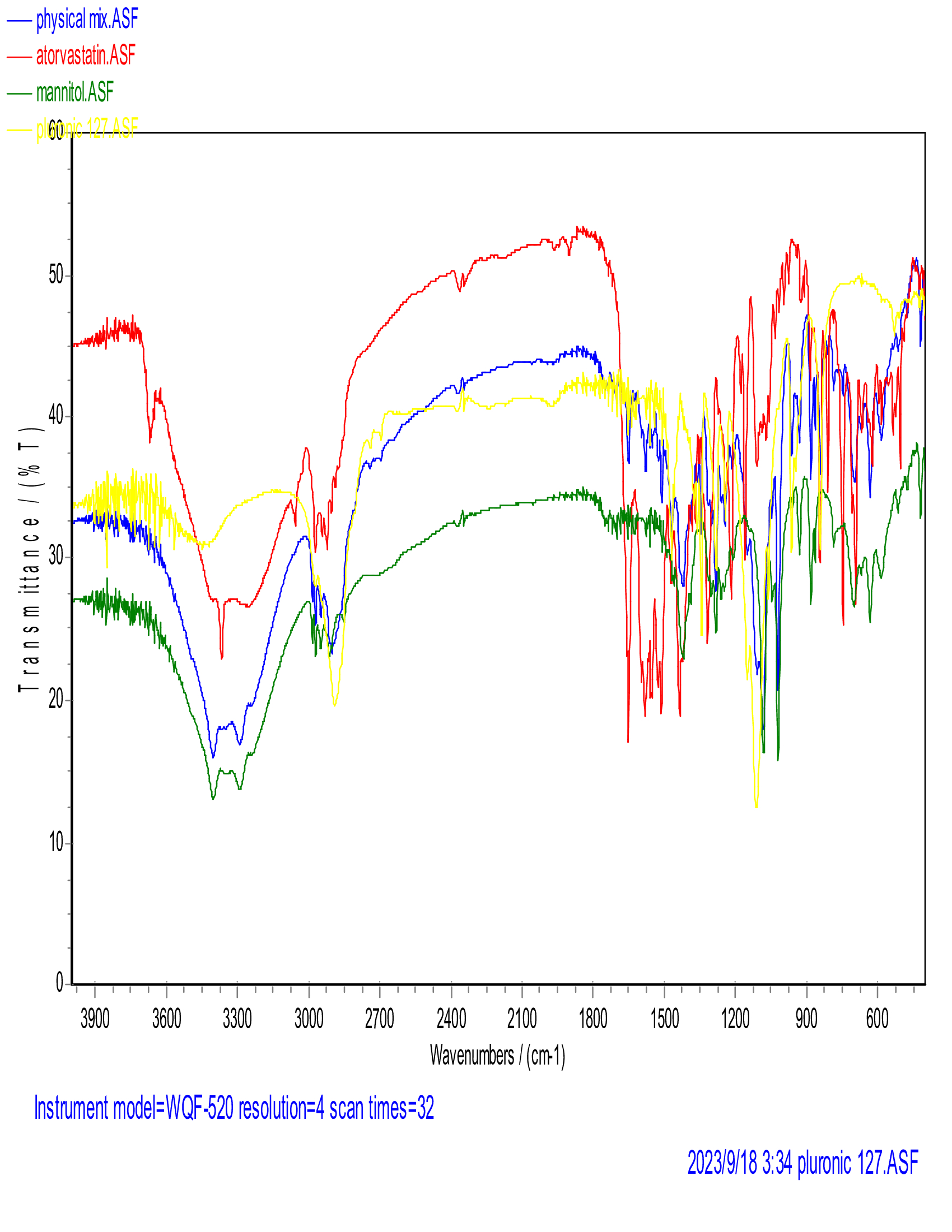

Supplement: S2 File — (ZIP) [file pone.0335024.s002.zip › FTIR data/pm_-mannitol_-atv_-pluronic.jpg]
